# No silver bullet: interpretable ML models must be explained

**DOI:** 10.3389/frai.2023.1128212

**Published:** 2023-04-24

**Authors:** Joao Marques-Silva, Alexey Ignatiev

**Affiliations:** ^1^IRIT, CNRS, Toulouse, France; ^2^Department of Data Science and Artificial Intelligence, Faculty of Information Technology, Monash University, Melbourne, VIC, Australia

**Keywords:** explainable AI (XAI), model interpretability, logic-based explainability, decision trees, decision lists, decision sets

## Abstract

Recent years witnessed a number of proposals for the use of the so-called interpretable models in specific application domains. These include high-risk, but also safety-critical domains. In contrast, other works reported some pitfalls of machine learning model interpretability, in part justified by the lack of a rigorous definition of what an interpretable model should represent. This study proposes to relate interpretability with the ability of a model to offer explanations of why a prediction is made given some point in feature space. Under this general goal of offering explanations to predictions, this study reveals additional limitations of interpretable models. Concretely, this study considers application domains where the purpose is to help human decision makers to understand why some prediction was made or why was not some other prediction made, and where irreducible (and so minimal) information is sought. In such domains, this study argues that answers to such why (or why not) questions can exhibit arbitrary redundancy, i.e., the answers can be simplified, as long as these answers are obtained by human inspection of the interpretable ML model representation.

## 1. Introduction

Recent years witnessed many successes of machine learning (ML) (LeCun et al., 2015; Goodfellow et al., 2016, 2020; Krizhevsky et al., 2017; Bengio et al., 2021). Despite these successes, there are shortcomings to the deployment of ML models (Szegedy et al., 2014; Goodfellow et al., 2015, 2016). Indeed, complex ML models can exhibit lack of robustness, can display bias, and their operation is invariably inscrutable for human decision makers (Gunning and Aha, 2019). As a result, there have been efforts to devising logically rigorous (and so formal) approaches to reasoning about ML models (Marques-Silva and Ignatiev, 2022).

In some application domains, e.g., in high-risk and safety-critical settings, a number of researchers have proposed the use of so-called interpretable models (Rudin, 2019; Molnar, 2020), which include, for example, decision trees, decision lists, and decision sets, among others (Molnar, 2020). Despite the term “*interpretable model*” being extremely popular (Rudin, 2019; Molnar, 2020), it is also the case that there is no rigorous definition for what an interpretable model should be. The subjectivity of what interpretability should mean indicates that a rigorous widely accepted definition is at least fairly unlikely. Accordingly, some other researchers have raised important concerns about what *interpretability* of ML models might represent (Lipton, 2018).

In the case of decision trees, we have recently shown (Izza et al., 2020, 2022a; Huang et al., 2021b) that, when compared with logically rigorous explanations, decision trees can yield explanations which are arbitrarily redundant on the number of features.^1^

Concretely, given some point in feature space and a predicted class, the question “why does the ML model predict the class?” is referred to as a WHY question. For decision trees, it has been shown (Izza et al., 2020, 2022a; Huang et al., 2021b) that the answer to this WHY question can be arbitrarily redundant when the explanation corresponds to the path in the decision tree that is consistent with the values assigned to the features. A corollary of these results is that, if succinct explanations can be viewed as a measure of model interpretability, then decision trees can hardly be deemed interpretable. Furthermore, a human decision maker will in most cases be unable to propose explanations less redundant than the tree path consistent with the input, and so automated computation of explanations is required.

This study extends further these earlier results on the redundancy of decision trees. We consider additional families of so-called interpretable ML models, and investigate what would be the answer to WHY questions. Since the internal details of the model are in general of no interest to a human decision maker, the answer to such a WHY question is to be expressed as an irreducible subset of the features, such that such set is *sufficient* for the prediction. A set of features is sufficient for the prediction if those features are fixed to their given values, then the value of the prediction must be the given one. Such definition enables *interpreting* the answers to WHY questions as logically correct universally valid rules, which can be conveyed to a human decision maker.

As with other related work, we seek answers to WHY questions which can be trusted. As a result, we need first to formalize what the answers to WHY questions mean. Afterwards, we argue that it is not intuitive (quite the contrary) to obtain such rigorous answers to WHY questions from (manual) inspection of the model. Thus, this further supports the argument against declaring ML model to be interpretable, even when these are claimed to be interpretable. The experimental results included in the study support extensively our conclusion. Concretely, the results show that explanations obtained by inspection of an ML model often include a significant degree of redundancy, and this represents information that is unnecessary to understand the reasons for why a prediction is being made.

Despite their shortcomings, there are still important reasons to advocate the use of these so-called interpretable models. One of these reasons is that such models can be efficiently explained in practice by using the rigorous definitions of explanations proposed in recent years. Given such definitions, we have provided empirical evidence that logically correct rules can be efficiently computed for several families of ML classifiers widely regarded as interpretable (Izza et al., 2020, 2022a; Huang et al., 2021b; Ignatiev and Marques-Silva, 2021). The assessment of the so-called interpretable models included in this study hinges on the fact that rigorous explanations are efficient to compute, even when in theory computing some of these explanations is computationally hard.

This article is organized as follows: Section 2 introduces the notations and definitions used throughout the article. Section 3 introduces logic-based explanations, and briefly overviews recent work on this topic. Section 4 proposes a measure of understanding ML models, namely model comprehensibility, and discusses examples that suggest that even interpretable models are not simple to comprehend. Section 5 summarizes a number of results which offer additional evidence to the difficulty in comprehending interpretable models. Section 6 analyzes experimental results on comprehending interpretable models, concretely decision trees and decision lists. The results of the article are briefly put into perspective in Section 7. Finally, the article concludes in Section 8.

## 2. Preliminaries

### 2.1. Classification problems

Classification problems in ML are defined on a set of features (or attributes) F={1,...,m} and a set of classes $K=\left\{c_{1},c_{2},...,c_{K}\right\}$. Each feature i∈F takes values from a domain 𝔻_*i*_. In general, domains can be categorical or ordinal, with values that can be boolean, integer, or real-valued. Feature space is defined as 𝔽 = 𝔻_1_ × 𝔻_2_ × . . . × 𝔻_*m*_. For boolean domains, 𝔻_*i*_ = {0, 1} = 𝔹, *i* = 1, . . . , *m*, and 𝔽 = 𝔹^*m*^. The notation **x** = (*x*_1_, . . ., *x*_*m*_) denotes an arbitrary point in feature space, where each *x*_*i*_ is a variable taking values from 𝔻_*i*_. The set of variables associated with features is *X* = {*x*_1_, . . ., *x*_*m*_}. Moreover, the notation **v** = (*v*_1_, . . ., *v*_*m*_) represents a specific point in feature space, where each *v*_*i*_ is a constant representing one concrete value from 𝔻_*i*_. When referring to the domains of one of more features, we use 𝔻 = 〈𝔻_1_, . . ., 𝔻_*m*_〉, which serves solely to aggregate all the features' domains in a inlinesingle dedicated structure.

With respect to the set of classes K, the size of K is assumed to be finite; no additional restrictions are imposed on K. An ML classifier M is characterized by a (non-constant) *classification function* *κ* that maps feature space 𝔽 into the set of classes K, i.e., κ:𝔽→K. An *instance* (or observation) denotes a pair (**v**, *c*), where **v** ∈ 𝔽 and c∈K, with *c* = *κ*(**v**). In should be plain to conclude that the formalization of ML classifiers imposes few (if any) restrictions on the families of classifiers that can be studied by using logic-based representations of those classifiers.

Given the definitions above, a classification problem is a tuple M=(F,𝔻,𝔽,K,κ), and 𝕄 denotes the set of all classification problems.

### 2.2. ML models regarded as interpretable

Although a wide range of ML models are often deemed interpretable (Molnar, 2020), we will consider tree and rule models (Flach, 2012), namely decision trees, decision lists, and decision sets, in their simplest forms. These are widely regarded as interpretable (Lakkaraju et al., 2016; Rudin, 2019, 2022; Molnar, 2020).

#### 2.2.1. Decision trees

**Figure 1:**
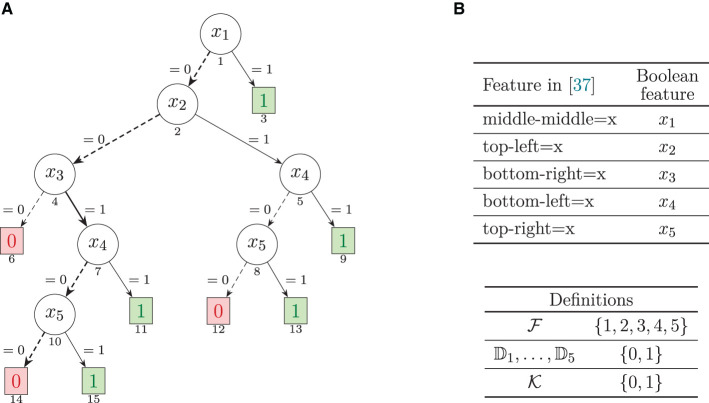
Decision tree, adapted from (Hu et al., 2019, Figure 5b), for the tic-tac-toe dataset. This DT is also studied more recently in Izza et al. (2022a) and Marques-Silva (2022). Each feature tests a possible play for the X player. The (boxed) terminal nodes display the predicted class. The (circled) non-terminal nodes display the tested feature. The edges label depicts the feature's tested literal, for the edge to be consistent. The number below each node denotes a unique number given to each node, which enables representing paths in the DT. **(A)** Decision tree. **(B)** Mapping of features.

A decision tree is a directed acyclic graph, with one root node that has no incoming edges, and the remaining nodes having exactly one incoming edge. Terminal nodes have no outgoing edges, and non-terminal nodes have two or more outgoing edges. Each terminal node is associated with a class, i.e., the predicted class for the node. Each non-terminal node is associated with exactly one feature (i.e., unless otherwise stated, we consider *univariate* DTs). Each outgoing edge is associated with a literal defined using the values of the feature, and such that any value of the feature domain is consistent with exactly one of the literals of the outgoing edges. In general, we allow literals to use the ∈ relational operator, as in earlier work (Izza et al., 2022a). Thus, a literal *x*_*i*_ ∈ {*S*_*i*_} is consistent if *x*_*i*_ takes one of the values in *S*_*i*_. For simplicity, when *S*_*i*_ = {*v*_*i*_}, then we will also allow for a literal to be of the form *x*_*i*_ = *v*_*i*_. Common (implicit) assumptions of DTs are that: i) all paths in a DT are consistent; and ii) the branches at each node capture all values in the domain of the tested feature. An example of a DT is shown in Figure 1. (This example will be analyzed in greater detail below).

#### 2.2.2. Decision lists and sets

Both decision lists and sets represent sets of rules. A rule is of the form: IF cond THEN class, i.e., if the condition cond is true given the values assigned to features, then class is predicted. When cond is true, we say that the rule *fires*; cond can for example represent a conjunction of literals, where a literal is defined as in the case of DTs. Moreover, the difference between decision rules and sets is that for decision lists, the rules are ordered, and for decision sets, the rules are unordered. Thus, a decision list is organized as follows:


(1) $$\begin{array}{l} R_{1}:\text{ IF }(\tau_{1})\text{ THEN }d_{1}\\ R_{2}:\text{ ELSE IF }(\tau_{2})\text{ THEN }d_{2}\\ \cdots \\ R_{r}:\text{ ELSE IF }(\tau_{r})\text{ THEN }d_{r}\\ {}[R_{DEF}:\text{ ELSE}\;d_{r+1}] \end{array}$$

In contrast, a decision set is organized as follows:


(2) $$\begin{array}{l} R_{1}:\text{ IF }(\tau_{1})\text{ THEN }d_{1}\\ R_{2}:\text{ IF }(\tau_{2})\text{ THEN }d_{2}\\ \cdots \\ R_{r}:\text{ IF }(\tau_{r})\text{ THEN }d_{r}\\ {}[R_{DEF}:\;\;d_{r+1}] \end{array}$$

Where the last rule is optional.

A difficulty with decision sets is rule overlap, i.e., the existence of situations when two or more rules predicting different classes fire (Observe that if overlapping rules predict different classes, then the decision set does not implement a classification function). Rule overlap was investigated in recent work (Lakkaraju et al., 2016), but with a definition of overlap that is restricted to the instances in the dataset. As a result, as first observed in Ignatiev et al. (2018), the solution proposed in Lakkaraju et al. (2016) is susceptible to overlap for points in feature space that are not in the dataset.

Another issue with decision sets (when a default rule is not used) is the fact that, for some points in feature space, it may be the case that no rule will fire. Approaches that guarantee no overlap were proposed in Ignatiev et al. (2018). To the best of our knowledge, no rigorous approach exists that guarantees that a decision sets implements a total function, i.e., guarantee of i) no overlap and ii) a prediction for every point in feature space. If these conditions are not met, interpretability is even more of a challenge (Furthermore, Ignatiev et al. (2018) conjectures that learning a DS that respects the two conditions above is ${\text{Σ}}_{2}^{\text{p}}$-hard).

The learning of decision sets that implement a total function without overlap is believed to be a computationally challenging task (Ignatiev et al., 2018). Moreover, no solution exists that guarantees that a decision set implements a total function without overlap. Thus, in the remainder of this article, we will focus on decision trees and decision lists.

### 2.3. Logic foundations

Throughout this article, we will use notations and definitions that are standard when reasoning about the decision problem for propositional logic, i.e., the Boolean Satisfiability (SAT) problem (Biere et al., 2021). SAT is well-known to be an NP-complete (Cook, 1971) decision problem. A propositional formula φ is defined over a finite set of propositional atoms *X* = {*x*_1_, *x*_2_, . . ., *x*_*n*_} (The elements of *X* are also referred to as boolean variables). Well-formed propositional formulas are defined inductively given a set of logic operators, ∧, ∨, and ¬ (resp. AND, OR, and NOT). Additionally often used logic operators include → and ↔ (resp. implication and equivalence). In practice, propositional formula are most often represented in *conjunctive normal form* (CNF). A CNF formula is a conjunction of clauses, a clause is a disjunction of literals, and a literal is a variable (*x*_*i*_) or its negation (¬*x*_*i*_). A term is a conjunction of literals. Whenever convenient, a formula is viewed as a set of sets of literals. A boolean interpretation (or valuation) ν of a formula *φ* is a total mapping of *X* to {0, 1} (0 corresponds to **false** and 1 corresponds to **true**). Interpretations can be extended to literals, clauses, and formulas with the usual semantics of propositional logic; hence, we can refer to *l*^ν^, *ω*^ν^, *τ*^ν^, and *φ*^ν^, to denote, respectively, the value assigned to a literal, clause, term, and formula, given an interpretation. Given a formula *φ*, ν is a *model* of *φ* if it makes *φ* **true**, i.e., *φ*^ν^ = 1. A formula *φ* is *satisfiable* (*φ* ⊭ ⊥) if it admits a model; otherwise, it is *unsatisfiable* (*φ* ⊨ ⊥). Given two formulas *φ* and *ψ*, we say that *φ* *entails* *ψ* (denoted *φ* ⊨ *ψ*) if all models of *φ* are also models of *ψ*. *φ* and *ψ* are equivalent (denoted *φ* ≡ *ψ*) if *φ* ⊨ *ψ* and *ψ* ⊨ *φ*.

For an unsatisfiable CNF formula *φ*, let T denote the set of clauses in *φ*. In this case, a *minimal unsatisfiable subset* (MUS) U is an irreducible subset of the clauses in T that is also unsatisfiable. A *minimal correction subset* (MCS) is an irreducible subset C of T, such that T\C is satisfiable. In general, these definitions can assume some background knowledge B, which is known to be consistent, and some other knowledge S, such that B∪S is unsatisfiable. A fundamental result in the analysis of inconsistent formulas is the minimal hitting set (MHS) duality between MUSes and MHSes (Reiter, 1987) (Recall that a set H is a *hitting set* of a set of sets $S=\left\{S_{1},...,S_{k}\right\}$ if $H\cap S_{i}\neq \emptyset$ for *i* = 1, . . ., *k*. H is a minimal hitting set of S, if H is a hitting set of S, and there is no proper subset of H that is also a hitting set of S). There exist in-depth overviews of algorithms for reasoning about inconsistent (or unsatisfiable) formulas, e.g., Marques-Silva and Mencía (2020).

## 3. Logic-based explainable AI

Logic-based (or formal) explanation approaches have been studied in a growing body of research in recent years (Shih et al., 2018, 2019; Ignatiev et al., 2019a,b,c, 2020a, 2022; Narodytska et al., 2019; Wolf et al., 2019; Audemard et al., 2020, 2021, 2022a,b; Boumazouza et al., 2020, 2021; Darwiche, 2020; Darwiche and Hirth, 2020, 2022; Izza et al., 2020, 2021, 2022a,b; Marques-Silva et al., 2020, 2021; Rago et al., 2020, 2021; Shi et al., 2020; Amgoud, 2021; Arenas et al., 2021; Asher et al., 2021; Blanc et al., 2021, 2022a,b; Cooper and Marques-Silva, 2021; Darwiche and Marquis, 2021; Huang et al., 2021a,b, 2022; Ignatiev and Marques-Silva, 2021; Izza and Marques-Silva, 2021, 2022; Liu and Lorini, 2021, 2022a; Malfa et al., 2021; Wäldchen et al., 2021; Amgoud and Ben-Naim, 2022; Ferreira et al., 2022; Gorji and Rubin, 2022; Huang and Marques-Silva, 2022; Marques-Silva and Ignatiev, 2022; Wäldchen, 2022; Yu et al., 2022), and are characterized by formally provable guarantees of rigor, given the underlying ML models. Given such guarantees of rigor, logic-based explainability should be contrasted with well-known model-agnostic approaches to XAI (Ribeiro et al., 2016, 2018; Lundberg and Lee, 2017; Guidotti et al., 2019), which offer no guarantees of rigor. The rest of this section offers a brief overview of logic-based explainability. More detailed overviews can be found elsewhere (Marques-Silva, 2022; Marques-Silva and Ignatiev, 2022).

### 3.1. Abductive explanations (AXp's)

Prime implicant (PI) explanations (Shih et al., 2018) denote a minimal set of literals (relating a feature value *x*_*i*_ and a constant *v*_*i*_ ∈ 𝔻_*i*_) that are sufficient for the prediction. PI explanations are related with abduction, and so are also referred to as abductive explanations (AXp's) (Ignatiev et al., 2019a)^2^. Formally, given **v** = (*v*_1_, . . ., *v*_*m*_) ∈ 𝔽 with *κ*(**v**) = *c*, a set of features X⊆F is a *weak abductive explanation* (or weak AXp) if the following predicate holds true^3^:


(3) $$\begin{array}{l} WeakAXp\left(X;\text{𝔽},\kappa ,\text{v},c\right):=\forall \left(\text{x}\in \text{𝔽}\right).\left[\wedge_{i\in X}\left(x_{i}=v_{i}\right)\right]\\ \;\to \left(\kappa \left(\text{x}\right)=c\right) \end{array}$$

Moreover, a set of features X⊆F is an *abductive explanation* (or (plain) AXp) if the following predicate holds true:


(4) $$\begin{array}{l} AXp\left(X;\text{𝔽},\kappa ,\text{v},c\right):=WeakAXp\left(X;\text{𝔽},\kappa ,\text{v},c\right)\wedge \\ \;\forall \left(X^{\prime }⊊X\right).\neg WeakAXp\left(X^{\prime };\text{𝔽},\kappa ,\text{v},c\right) \end{array}$$

Clearly, an AXp is any weak AXp that is subset-minimal (or irreducible). It is straightforward to observe that the definition of predicate WeakAXp is monotone, and so an AXp can instead be defined as follows:


(5) $$\begin{array}{l} AXp\left(X;\text{𝔽},\kappa ,\text{v},c\right):=WeakAXp\left(X;\text{𝔽},\kappa ,\text{v},c\right)\wedge \\ \;\forall \left(j\in X\right).\neg WeakAXp\left(X\backslash \left\{j\right\};\text{𝔽},\kappa ,\text{v},c\right) \end{array}$$

This alternative equivalent definition of abductive explanation is at the core of most algorithms for computing one AXp (Throughout the article, we will drop the parameterization associated with each predicate, and so we will write AXp(X) instead of AXp(X;𝔽,κ,v,c), when the parameters are clear from the context).

Example 1. We consider the example decision tree from Figure 1, and the instance (**v**, *c*) = ((0, 0, 1, 0, 1), 1). By inspection (or by following the discussion in Izza et al., 2022a), we can conclude that {3, 5} is the only AXp, given the instance.

It is apparent that (3), (4), and (5) can be viewed as representing a (logic) *rule* of the form:


(6) $$\begin{array}{l} \text{IF}\underset{i\in X}{\wedge }\left(x_{i}=v_{i}\right)\text{THEN}\;\kappa \left(\text{x}\right)=c \end{array}$$

This interpretation of abductive explanations will be assumed throughout the article.

Similar to non-formal approaches to explainability (Ribeiro et al., 2018), abductive explanations can be interpreted as answering a “**WHY**” question, i.e., why is some prediction made given some point in feature space. The answer to this question is a (minimal or irreducible) set of the features, which is sufficient for (or entails) the prediction.

### 3.2. Contrastive explanations (CXp's)

Similarly to the case of AXp's, one can define (weak) contrastive explanations (CXp's) (Miller, 2019; Ignatiev et al., 2020a).^4^ Y⊆F is a weak CXp for the instance (**v**, *c*) if,


(7) $$\begin{array}{l} WeakCXp\left(Y;\text{𝔽},\kappa ,\text{v},c\right):=\exists \left(\text{x}\in \text{𝔽}\right).\left[\wedge_{i\notin Y}\left(x_{i}=v_{i}\right)\right]\\ \;\wedge \left(\kappa \left(\text{x}\right)\neq c\right) \end{array}$$

(As before, for simplicity, we will often keep the parameterization of WeakCXp on *κ*, **v**, and *c* implicit). Thus, given an instance (**v**, *c*), a (weak) CXp is a set of features which if allowed to take any value from their domain, then there is an assignment to the features that changes the prediction to a class other than *c*, while the features not in the explanation are kept to their values (*ceteris paribus*). Furthermore, a set Y⊆F is a CXp if, besides being a weak CXp, it is also subset-minimal, i.e.,


(8) $$\begin{array}{l} CXp\left(Y;\text{𝔽},\kappa ,\text{v},c\right):=WeakCXp\left(Y;\text{𝔽},\kappa ,\text{v},c\right)\wedge \\ \;\forall \left(Y^{\prime }⊊Y\right).\neg WeakCXp\left(Y^{\prime };\text{𝔽},\kappa ,\text{v},c\right) \end{array}$$

Similar to the case of AXp's, it is straightforward to observe that the definition of predicate WeakCXp is monotone, and so an CXp can instead be defined as follows:


(9) $$\begin{array}{l} CXp\left(Y;\text{𝔽},\kappa ,\text{v},c\right):=WeakCXp\left(Y;\text{𝔽},\kappa ,\text{v},c\right)\wedge \\ \;\forall \left(t\in Y\right).\neg WeakCXp\left(Y\backslash \left\{t\right\};\text{𝔽},\kappa ,\text{v},c\right) \end{array}$$

Moreover, and again similar to the case of AXp's, this simplified definition of CXp is at the core of algorithms for their computation.

A key observation is that *any* solution of (7), (8), or (9) (be it minimal or not) identifies not only a set non-fixed of features but also assignments to those non-fixed features that guarantee a change of the prediction. Hence, all the information required to change the prediction is readily available. Furthermore, the definition of CXp (similar to the definition of AXp) targets a subset-minimal set of features. However, other definitions could be considered, e.g., cardinality-minimal contrastive explanations, among others.

Example 2. We consider the example decision tree from Figure 1, and the instance (**v**, *c*) = ((0, 0, 1, 0, 1), 1). By inspection (or by following the discussion in Izza et al., 2022a), we can conclude that {3} and {5} are the only CXp's, given the instance.

A CXp can be viewed as a possible answer to a “**WHYNOT**” question, i.e., “why is not the classifier's prediction a class other than *c*?” (Clearly, the definition can be adapted to the case when we seek a concrete change of class.) A different perspective for a contrastive explanation is the answer to a “**How?**” question, i.e., how to change the features so as to change the prediction. In recent literature, this alternative view has been investigated under the name “*actionable recourse*” (Ustun et al., 2019; Karimi et al., 2020, 2021; Venkatasubramanian and Alfano, 2020).

### 3.3. Duality between AXp's and CXp's

Given the definitions of AXp and CXp, and building on Reiter's seminal work (Reiter, 1987) (see Section 2.3), recent work (Ignatiev et al., 2020a,b) proved the following duality between minimal hitting sets:

Proposition 1. (Minimal hitting-set duality between AXp's and CXp's Ignatiev et al., 2020a,b) AXp's are minimal hitting sets (MHSes) of CXp's and *vice versa*.

We refer to Proposition 1 as MHS duality between AXp's and CXp's.

Example 3. We consider the DT running example from Figure 1, and the instance, (**v**, *c*) = ((0, 0, 1, 0, 0), 0). Once more by inspection (or by following the discussion in Izza et al., 2022a), we can conclude that the sets of AXp's is: {{1, 4, 5}} and that the set of CXp's is {{1}, {4}, {5}}.

Proposition 1 has been used in more recent work for enabling the enumeration of explanations (Huang et al., 2021b; Ignatiev and Marques-Silva, 2021; Marques-Silva et al., 2021).

### 3.4. Current status of logic-based explainability

There has been steady progress in the efficient computation of explanations (Marques-Silva, 2022; Marques-Silva and Ignatiev, 2022) (and references therein). Moreover, a number of related research topics have been investigated, including enumeration of explanations (Ignatiev et al., 2020a), explainability queries (Audemard et al., 2020, 2021; Huang et al., 2021b), probabilistic explanations (Wäldchen et al., 2021; Arenas et al., 2022; Izza et al., 2022b), or taking into account constraints in feature space (Gorji and Rubin, 2022; Yu et al., 2022). For the purposes of this article, the more important results are^5^:

1. For decision trees, there are polynomial-time algorithms for computing one AXp, all CXp's can be computed in polynomial time, and there are practically efficient algorithms for the enumeration of AXp's (Izza et al., 2020, 2022a; Huang et al., 2021b).
2. For decision lists, it is computationally hard to compute one AXp/CXp, but existing logic encodings enable the practically efficient computation of one explanation and of the enumeration of explanations (Ignatiev and Marques-Silva, 2021).
3. The approach used for decision lists can also be used in the case of decision sets (Ignatiev and Marques-Silva, 2021), but here the main limitation is requiring that the decision set computes a total function, as discussed earlier in this article.

## 4. How to understand interpretable ML models?

Since there is no formal definition of what interpretability means, and since such a definition is unlikely (Lipton, 2018), we ask a different question. Concretely, this section investigates how explanations can be obtained from an interpretable model. Since the model is interpretable, we require that a human decision maker be able to find such an explanation by *manual inspection*, i.e., not automated analysis is to be used. Evidently, for an interpretable model, one would expect that this should be feasible to do.

### 4.1. How to comprehend predictions?

A natural first question is how can a human decision maker understand predictions. Following earlier work (Miller, 2019; Molnar, 2020), we investigate how explanations can be manually identified given an interpretable model. Concretely, given some interpretable model, e.g., decision trees, lists, or sets, we pose the following question:

“Given an instance (**v**, *c*), why is the prediction *c*?”

We refer to this question as the WHY question. Similar to recent work on non-formal interpretability and explainability (Lakkaraju et al., 2016; Ribeiro et al., 2018), we seek to answer the WHY question by finding a set of features X, with which we associate the following rule:


(10) $$\begin{array}{lll} \text{Xp}:\;\text{IF}\wedge_{i\in X}\left(x_{i}=v_{i}\right) & \text{THEN} & \kappa \left(\text{x}\right)=c \end{array}$$

Clearly, this rule is required to be logically correct. Moreover, Occam's razor is expected of X, i.e., we require X to be irreducible (i.e., subset-minimal) (Even if one ensures irreducibility, a possible criticism is that if the size of X is too large, then the answer to the WHY question may be beyond the cognitive grasp of a human decision maker (Miller, 1956). Methods to address this possible limitation have been studied elsewhere (Wäldchen et al., 2021; Arenas et al., 2022; Izza et al., 2022b)). Furthermore, to keep the notation as simple as possible, and similarly to the definition of AXp's and CXp's, we will talk about the answers to the WHY questions solely using only sets of features. Concretely, X⊆F is a set of features, that presupposes a literal (*x*_*i*_ = *v*_*i*_) for each i∈X. As a result, the relationship of a set of features X with the rule above is immediate.

Another extensively studied type of explanation is contrastive (often referred to as counterfactual) explanations. Similarly to the case of WHY questions, given some interpretable model, e.g., decision trees, lists, or sets, we pose the following question:

“Given an instance (**v**, *c*) why is the prediction not a class other than *c*?”

Put another way, what should be changed to change the prediction? We refer to this question as the WHYNOT question.

### 4.2. Defining model comprehensibility

As a measure of the actual interpretability of an ML model, we propose instead the concept of model comprehensibility. Concretely, we say that an (interpretable) ML model is comprehensible if:

The ML model enables a human decision maker, *via* non-automated analysis (i.e., by manual inspection of the model), to rigorously answer a WHY question, thereby finding a set of features that is both sufficient for the prediction and irreducible.

Clearly, for interpretable ML models [i.e., those where the explanation is the model itself (Rudin, 2019; Molnar, 2020)], one would expect the model to be comprehensible, thus enabling a human decision maker to grasp answers to the WHY question, and express such answers as general rules, as proposed above. As shown in the rest of this section, although one can devise solutions for finding correct answers to the WHY question, those answers are hardly irreducible. More importantly, as shown in later sections, arbitrary redundancy is inherent to models that are generally deemed interpretable.

It should be observed that an answer to the WHY question corresponds ideally to an AXp^6^. Hence, if we can find AXp's, then we can provide answers to the WHY questions. Similarly, an answer to the WHYNOT questions corresponds ideally to a CXp. Given the above, we can thus conclude that what we are interested, essentially, to assess whether manual analysis of an interpretable model by a human decision maker will serve to find AXp's and/or CXp's. As will be clarified later in the article (see Section 5.4), manually identifying answers to WHYNOT questions can be substantially more difficult that identifying answers to WHY questions.

### 4.3. Are interpretable models indeed comprehensible?

Before delving into theoretical results on ML model comprehensibility, let us motivate such results with a number of examples. We will analyze decision trees and decision lists, seeking to propose correct approaches for computing answers to the WHY question. Furthermore, we will also inquire how realistic it is to find answers that are both correct and irreducible.

#### 4.3.1. Decision trees

For a decision tree, an intuitive manual approach to propose an answer to the WHY question is as follows (Izza et al., 2022a):

1. Pick the features with literals in the path that is consistent with the prediction.

Clearly, such picked set of features implies a rule that is correct. However, it is unclear whether the set of features is irreducible.

Example 4. For the example in Figure 1, let the instance be **v** = (0, 0, 1, 0, 1). Thus, an answer to the WHY question would be X={1,2,3,4,5}, thus capturing the rule,


$$\begin{array}{cccc} \text{Xp}:\;\text{IF} & \left[\left(\neg x_{1}\right)\wedge \left(\neg x_{2}\right)\wedge \left(x_{3}\right)\wedge \left(\neg x_{4}\right)\wedge \left(x_{5}\right)\right] & \text{THEN} & \kappa \left(\text{x}\right)=1 \end{array}$$

It is not too difficult to understand what X is not irreducible. For example, if we allow *x*_1_ to change value, then the prediction will remain unchanged; hence $X^{\prime }=\left\{2,3,4,5\right\}$ is also an answer to the WHY question. However, one can provide a much shorter answer. Let us allow features 1, 2, and 4 to take any value, with *x*_3_ = *x*_5_ = 1, and let us check the predicted values. The result of this exercise is shown in Table 1. As can be observed, since the prediction remains unchanged for any value assigned to features 1, 2, and 4, we can conclude that an answer to the WHY question in this case is $X^{\prime \prime }=\left\{3,5\right\}$. It is also fairly simple to conclude that $X^{\prime \prime }$ is indeed irreducible. However, it seems apparent that most human decision makers would be unable to fathom $X^{\prime \prime }$ by inspection of the decision tree.

**Table 1:** Considering all possible values of *x*_1_, *x*_2_, *x*_4_ when *x*_3_ = *x*_5_ = 1.

***x*_3_ = *x*_5_**	***x*_1_**	***x*_2_**	***x*_4_**	**κ(x)**
1	0	0	0	1
1	0	0	1	1
1	0	1	0	1
1	0	1	1	1
1	1	0	0	1
1	1	0	1	1
1	1	1	0	1
1	1	1	1	1

One might also wonder whether one should be interested in irreducible answers to the WHY question. As illustrated by this example, one would expect that the average human decision maker will be able to relate far better with the following (irreducible) rule,


$$\begin{array}{cccc} \text{Xp}:\;\text{IF} & \left[\left(x_{3}\right)\wedge \left(x_{5}\right)\right] & \text{THEN} & \kappa \left(\text{x}\right)=1 \end{array}$$

than with the rule that includes all five features (shown above).

#### 4.3.2. Decision lists

For decision lists, we can raise similar questions. As argued below, answering WHY questions may not be immediate.

Example 5. Consider a DL classifier, with *κ*(*x*_1_, *x*_2_, *x*_3_, *x*_4_, *x*_5_) defined by,


(11) $$\begin{array}{l} \begin{array}{cccccc} {\text{R}}_{1}: & \; & \text{IF} & \left(\neg x_{1}\wedge \neg x_{2}\right) & \text{THEN} & \kappa \left(\text{x}\right)=0\\ {\text{R}}_{2}: & \; & \text{ELSE IF} & \left(x_{1}\wedge x_{2}\wedge \neg x_{3}\right) & \text{THEN} & \kappa \left(\text{x}\right)=1\\ {\text{R}}_{3}: & \; & \text{ELSE IF} & \left(x_{1}\wedge x_{2}\wedge x_{3}\wedge x_{4}\wedge x_{5}\right) & \text{THEN} & \kappa \left(\text{x}\right)=1\\ {\text{R}}_{4}: & \; & \text{ELSE IF} & \left(x_{1}\wedge x_{2}\wedge \neg x_{4}\right) & \text{THEN} & \kappa \left(\text{x}\right)=1\\ {\text{R}}_{5}: & \; & \text{ELSE IF} & \left(x_{1}\wedge x_{2}\wedge \neg x_{5}\right) & \text{THEN} & \kappa \left(\text{x}\right)=1\\ {\text{R}}_{\text{DEF}}: & \; & \text{ELSE} & \; & \; & \kappa \left(\text{x}\right)=0 \end{array} \end{array}$$

Let **v** = (1, 1, 1, 1, 1). Clearly, *κ*(**v**) = 1.

Suppose we are interested in answering the question: “*Why is the prediction 1 for* **v** = (1, 1, 1, 1, 1)*?*”. Since the model is interpretable, we seek an answer by inspection of the DL. A possible answer is X={1,2,3,4,5}, i.e., as long as all the features take the value in **v**, then it is certainly the case that the prediction is 1. However, if the user seeks shorter (logically) correct explanations, e.g., that contain no redundant information, then it may be possible to offer the user far more insightful information (Nevertheless, it should be noted that obtaining such information requires some degree of logical reasoning, which may not be immediate for the average human decision maker). For the example above, it can be shown that X={1,2} is a logically correct explanation, i.e., as long as *x*_1_ = *x*_2_ = 1, then the prediction will be 1, *independently* of the values taken by the other features.

Example 6. Even if some human decision maker can understand why the answer to the WHY question is X={1,2} for the DL above, more subtle scenarios can be be envisioned. Let us consider the following DL:


(12) $$\begin{array}{l} \begin{array}{cccccc} {\text{R}}_{1}: & \; & \text{IF} & \left(x_{1}\wedge x_{3}\right) & \text{THEN} & \kappa \left(\text{x}\right)=1\\ {\text{R}}_{2}: & \; & \text{ELSE IF} & \left(x_{2}\wedge x_{4}\wedge x_{6}\right) & \text{THEN} & \kappa \left(\text{x}\right)=0\\ {\text{R}}_{3}: & \; & \text{ELSE IF} & \left(\neg x_{1}\wedge x_{3}\right) & \text{THEN} & \kappa \left(\text{x}\right)=1\\ {\text{R}}_{4}: & \; & \text{ELSE IF} & \left(x_{4}\wedge x_{6}\right) & \text{THEN} & \kappa \left(\text{x}\right)=0\\ {\text{R}}_{5}: & \; & \text{ELSE IF} & \left(\neg x_{1}\wedge \neg x_{3}\right) & \text{THEN} & \kappa \left(\text{x}\right)=1\\ {\text{R}}_{6}: & \; & \text{ELSE IF} & \left(x_{6}\right) & \text{THEN} & \kappa \left(\text{x}\right)=0\\ {\text{R}}_{\text{DEF}}: & \; & \text{ELSE} & \; & \; & \kappa \left(\text{x}\right)=1 \end{array} \end{array}$$

Let the point in feature space be **v** = (0, 1, 0, 1, 0, 1), with *κ*(**v**) = 0, i.e., rule R_2_ fires. If a human decision maker is interested in answering the question: “*Why is the prediction 0 for* **v** = (0, 1, 0, 1, 0, 1)*?*”, what are possible explanations from inspecting the model? One might be tempted to state that if *x*_2_ = *x*_4_ = *x*_6_ = 1, then the prediction is 0, i.e., the explanation is the condition of R_2_. However, such answer is incorrect. For example, if both *x*_1_ and *x*_3_ are flipped to 1, then the prediction would become 1, due to R_1_ firing; this means that *x*_2_ = *x*_4_ = *x*_6_ = 1 is not a correct explanation. A possible solution is to weaken the explanation, by including additional literals. For example, if either *x*_1_ or *x*_3_ are 0 then, if *x*_2_ = *x*_4_ = *x*_6_ = 1, it is the case that the prediction is 0. So, a possible explanation is *x*_3_ = 0 and *x*_2_ = *x*_4_ = *x*_6_ = 1. Does this explanation represent an irreducible set of literals? Unsurprisingly, the answer is no, and a more careful analysis allows concluding that the answer to the WHY question is: *x*_3_ = 0 and *x*_4_ = *x*_6_ = 1. It should be apparent from the previous example, that even for simple DLs, finding an answer to a WHY question, which is both correct and irreducible, is not a trivial task. With the purpose of highlighting the challenges of finding correct and irreducible answers to WHY questions, let us consider again the decision list in (12), and let the point in feature space be **v** = (0, 0, 0, 0, 0, 0), with *κ*(**v**) = 0. In this case, since the default rule fires, there is no condition of the rule to start from. Building on the examples above, one might propose *x*_3_ = *x*_5_ = *x*_6_ as an explanation. However, more careful analysis confirms that *x*_6_ = 0 suffices as an (irreducible) answer to a WHY question, i.e., if *x*_6_ = 0, then the prediction will be 1 independently of the values of all the other features. Somewhat less intuitive might be that *x*_1_ = *x*_4_ = 0 which is also an irreducible answer to a WHY question, i.e., if *x*_1_ = *x*_4_ = 0, then the prediction will be 1 independently of the values of all the other features.

The previous example highlighted some of the requirements for manually answering a WHY question in the case of a decision list. Consider the definition of decision list in (1). Pick some instance (**v**, *c*). Let R_*j*_ denote the rule that fires, and let the prediction be *c*. Hence, we propose to find a correct answer A⊆F to the WHY question as follows:

1. All the features associated with literals in *τ*_*j*_ are added to A;
2. For each rule R_*k*_ preceding R_*j*_, that predicts a class other than *c*, let *i* be the feature of the first literal inconsistent with **v**. Then add feature *i* to A.

We could conceivably propose optimizations to the procedure above, but these would make it far more difficult for a human decision maker to find on his/her own answers to the WHY questions. However, as illustrated by the next example, that would still not guarantee that the computed answer would be irreducible.

Example 7. Finally, let us consider the following DL:


(13) $$\begin{array}{l} \begin{array}{cccccc} {\text{R}}_{1}: & \; & \text{IF} & \left(x_{1}\wedge x_{3}\right) & \text{THEN} & \kappa \left(\text{x}\right)=0\\ {\text{R}}_{2}: & \; & \text{ELSE IF} & \left(x_{1}\wedge x_{5}\right) & \text{THEN} & \kappa \left(\text{x}\right)=0\\ {\text{R}}_{3}: & \; & \text{ELSE IF} & \left(x_{2}\wedge x_{4}\right) & \text{THEN} & \kappa \left(\text{x}\right)=1\\ {\text{R}}_{4}: & \; & \text{ELSE IF} & \left(x_{1}\wedge x_{7}\right) & \text{THEN} & \kappa \left(\text{x}\right)=0\\ {\text{R}}_{5}: & \; & \text{ELSE IF} & \left(\neg x_{4}\vee x_{6}\right) & \text{THEN} & \kappa \left(\text{x}\right)=1\\ {\text{R}}_{6}: & \; & \text{ELSE IF} & \left(\neg x_{4}\vee \neg x_{6}\right) & \text{THEN} & \kappa \left(\text{x}\right)=1\\ {\text{R}}_{7}: & \; & \text{ELSE IF} & \left(\neg x_{2}\vee x_{6}\right) & \text{THEN} & \kappa \left(\text{x}\right)=1\\ {\text{R}}_{\text{DEF}}: & \; & \text{ELSE} & \; & \; & \kappa \left(\text{x}\right)=0 \end{array} \end{array}$$

with **v** = (0, 1, 0, 1, 0, 1, 0). Clearly, the prediction is 1, due to R_3_. What should be the answer to a WHY question in this case? Given what we discussed until now, we might be tempted to propose X={1,2,4}, since fixing feature 1 will prevent rules R_1_ and R_2_ from firing, and fixing features 2 and 4 will ensure that rule R_3_ fires. However, since fixing feature 1 also prevents rule R_4_ from firing, then the value of feature 4 is actually irrelevant, since *x*_4_ = 0 would cause rule R_5_ to fire, with *x*_1_ = 0 and *x*_2_ = 1. Thus, an irreducible answer to the WHY question should be X={1,2}. The point here is that to find a subset minimal explanation we must not only consider the rules that precede the rule that fired, but also the rules that follow the rule that fired. As before, it appears unrealistic that the average human decision maker would grasp the answer {1, 2} by inspection of the DL. More importantly, and similarly to earlier examples, this is a rather simple example: one should expect far more complex examples in practice. Observe that {1} does not suffice as the justification for why the prediction is 1. Indeed, by allowing **u** = (0, 0, 0, 1, 0, 0, 0) would cause the prediction to change to 0 due to the default rule. Hence, feature 2 is necessary for preventing the prediction from changing.

As proved elsewhere (Ignatiev and Marques-Silva, 2021), it is hard to compute one AXp (resp. CXp) in the case of DLs/DSs. Hence, it would be unrealistic to expect human decision makers to be able to compute answers to the WHY (resp. WHY NOT) question by inspection, as each such answer can be mapped to an AXp (resp. CXp).

## 5. Non-comprehensibility of interpretable models

Given the understanding of comprehensibility proposed in the previous section, we now argue that even the simplest ML models, which are ubiquitously deemed interpretable, do not respect such understanding. The ensuing conclusion is that, for finding answers to the WHY question, even so-called interpretable models should be explained, by using a rigorous definition of explanation as proposed in Section 3.

### 5.1. Non-comprehensibility of decision trees

This section summarizes recent results on the non-comprehensibility of DTs. The underlying assumption is that the answer for a WHY question is the path consistent with the values of the features.

Proposition 2. (Corollary 2 of Izza et al., 2022a) There exist DTs, defined on *m* features, for which there exist instances exhibiting an AXp of size 1, and the path consistent with the instances has size *m*.

Proposition 3. (Proposition 11 of Izza et al., 2022a) A DT does not exhibit explanation redundancy if it can be represented with a disjunctive normal form (DNF) generalized decision function (GDF).

A GDF is a very restricted class of classifier, and so Proposition 3 indicates that the class of functions that can be represented with DTs without exhibiting path explanation redundancy is very restricted.

### 5.2. Non-comprehensibility of decision lists

The examples analyzed in Section 4 suggest that a straightforward approach for answering WHY questions in DLs is bound to yield explanations that contain redundant literals.

As experimentally validated by the results in Section 6, this is indeed the case.

Clearly, one might argue that a different approach for finding explanations would yield less or no redundancy. We conjecture that for any manual approach, producing explanations will necessarily introduce redundancy. Concretely, for any pre-specified approach for computing explanations by hand, one can construct a DL for which explanations will exhibit redundancy.

### 5.3. Non-comprehensibility of decision sets

Previous sections highlighted the many issues with DSs in practical settings. If a DS does not compute a function, then the core assumptions of logic-based explainability are not respected. If the DS computes a partial function, then again the core assumptions of logic-based explainability are not respected. One additional hurdle is that the learning of DSs that compute total functions (and so ensure that no overlap exists) is conjectured to be $\Sigma_{2}^{\text{p}}$-hard (Ignatiev et al., 2018). Finally, explanation of DSs when these compute total functions appears to raise at least the same difficulties as DLs.

### 5.4. Answering WHYNOT questions can be hard

In stark contrast with finding correct answers to WHY questions in DLs, this section proves that the apparently trivial problem of answering a WHYNOT question for a DL is NP-complete, i.e., it is computationally hard to decide whether the prediction can be changed to some other class. The implication of this result is that it would be rather unrealistic to expect human decision makers to decide NP-complete problems when proposing answers to WHYNOT questions. The implication of this result is that interpretability is unattainable when the goal is to answer WHYNOT questions for DLs.

Throughout this section, we consider a CNF formula *φ*, defined on a set of propositional atoms {*x*_1_, . . ., *x*_*m*_}, composed of clauses {ς_1_, . . ., ς_*n*_}, such that each clause ς_*i*_ contains three literals and it is non-tautologous. Assignments map each atom to {0, 1}. Given an assignment, the valuation of a CNF formula *φ* maps *φ* to {0, 1} (Biere et al., 2021). The decision problem for CNF formulas (i.e., the Boolean Satisfiability (SAT) problem) is to decide whether there exists an assignment such that the formula's valuation is 1. It is well-known that SAT is NP-complete (Cook, 1971) (Technically, for CNF formulas, the decision problem is CNFSAT, and since we consider each clause to contain three literals, the decision problem is referred to as 3CNFSAT. However, we just use SAT to refer to these as well as the original decision problem on arbitrary propositional formulas).

** Definition 1 (TOGGLESOME)**. Given a CNF formula *φ*, and given an assignment to the atoms of *φ* that falsifies at least one clause of *φ*, decide the satisfiability of *φ*.

Proposition 4. TOGGLESOME is NP-complete.

Proof.(Sketch) TOGGLESOME is in NP. We ignore the starting assignment, guess an assignment to the variables of *φ*, and then check in polynomial time whether *φ* takes value 1 given the assignment.

To prove NP-hardness, we reduce SAT to TOGGLESOME. Pick a clause ς in *φ*, and falsify it. For the remaining atoms, pick a random assignment. Thus, *φ* with its falsified clause ς (and with other clauses also possibly falsified) and the picked assignment represent an instance of TOGGLESOME. Clearly, *φ* is satisfiable if and only if the answer to TOGGLESOME is positive, and the reduction runs in polynomial time.

In the case of a DL, we are interested in the following generic problem. Given an instance (**v**, *c*), can **v** be modified so that the prediction is $c^{\prime }\in K\backslash 8\{c\}$? As shown next, just deciding whether a prediction can be changed is in fact a computationally hard problem.

Proposition 5. Deciding whether a prediction in a DL can be changed is NP-complete.

Proof. We consider a DL, such that rule *j* with prediction *c* fired on some input. We want to decide which features to change such that the prediction changes value to a class other than *c*.

The problem is clearly in NP. We non-deterministacilly guess a certificate, i.e., an assignment of values to the features, and then check whether the resulting prediction is different than the starting one.

To prove NP-hardness, we reduce TOGGLESOME to the problem of deciding the existence of a set of features which, if changed, will allow changing the prediction.

Let *φ* be a CNF formula (as described above). Without loss of generality, we consider a renumbering of the clauses of *φ*, such that the picked assignment falsifies the first clause; let it be ς_1_, with the three literals of ς_1_ referenced by *l*_1_(ς_1_), *l*_2_(ς_1_), and *l*_3_(ς_1_). Moreover, since the clauses are non-tautologous, then ¬ς_*i*_ is a non-inconsistent conjunction of propositional literals for any clause ς_*i*_ of *φ*. Now, we construct the following decision list:


$$\begin{array}{cccccc} {\text{R}}_{1}: & \; & \text{IF} & z\wedge \neg \varsigma_{1} & \text{THEN} & 0\\ {\text{R}}_{2}: & \; & \text{ELSE IF} & z\wedge \neg \varsigma_{2} & \text{THEN} & 0\\ & & & \cdots \; & & \\ {\text{R}}_{n}: & \; & \text{ELSE IF} & z\wedge \neg \varsigma_{n} & \text{THEN} & 0\\ {\text{R}}_{n+1}: & \; & \text{ELSE IF} & z & \text{THEN} & 1\\ {\text{R}}_{n+2}: & \; & \text{ELSE IF} & \neg z\wedge \neg \varsigma_{1} & \text{THEN} & 0\\ {\text{R}}_{n+3}: & \; & \text{ELSE IF} & l_{1}\left(\varsigma_{1}\right) & \text{THEN} & 0\\ {\text{R}}_{n+4}: & \; & \text{ELSE IF} & l_{2}\left(\varsigma_{1}\right) & \text{THEN} & 0\\ {\text{R}}_{n+5}: & \; & \text{ELSE IF} & l_{3}\left(\varsigma_{1}\right) & \text{THEN} & 0\\ {\text{R}}_{\text{DEF}}: & \; & \text{ELSE} & \; & \text{THEN} & 1 \end{array}$$

where *z* is a fresh propositional variable.

Furthermore, we consider some input that causes *R*_*n*+2_ to fire, resulting in prediction 0. Then, it must be the case that *z* = 0 and that ς_1_ is falsified. Now, for the prediction to change, rules R_1_, . . ., R_*n*_ must not fire, and rule R_*n*+1_ must fire. Since we must have *z* = 1 for R_*n*+1_ to fire, then each ¬ς_*i*_ must be falsified (and so each ς_*i*_ must be satisfied). As a result, the prediction changes to 1 if and only if *φ* is satisfied, i.e., that the answer to TOGGLESOME is positive. Observe that the alternative to change the prediction would be for R_DEF_ to fire. That would require both *z* = 0 and each literal of ς_1_ to be falsified; but then rule R_*n*+2_ would still fire before the default rule, and that would mean no change in the prediction. Hence, it is impossible for R_DEF_ to fire, and so a change of prediction requires R_*n*+1_ to fire.

(It should be observed that the construction used in rules R_*n*+3_, R_*n*+4_, and R_*n*+5_, which render R_DEF_ unreachable, is by no means restrictive. First, the reduction is still from some CNF formula (as an instance of TOGGLESOME) to a DL. Second, a more involved reduction could have been proposed instead. A solution would be to reduce *φ*_1_ ∨ *φ*_2_, where *φ*_1_ would be encoded into rules R_1_, . . ., R_*n*_1__, and *φ*_2_ would be encoded into rules R_*n*_1_+3_, …, R_*n*_1_+*n*_2_+1_. In addition, R_*n*+2_ would consist of *z*∧¬ς_11_∧¬ς_21_, requiring at least one clause of *φ*_1_ to be falsified and at least one clause of *φ*_2_ to be falsified. It should be plain that the geralization of TOGGLESOME to the case of *φ*_1_ ∨ *φ*_2_ is straightforward).

Observe that answering a WHYNOT question amounts to deciding whether the prediction can be changed, and that is NP-complete as proved above. Intuitively, the complexity of finding one CXp results from the need to make consistent the condition of some rule R_*k*_, that predicts some class other than *c*, and such that all the rules that precede R_*k*_ must be inconsistent.

It should be noted that the result above could also be established by relating with earlier results on the complexity of computing explanations for DLs (Ignatiev and Marques-Silva, 2021, Prop. 3) and the relationship between the complexity of computing AXp's and CXp's (Cooper and Marques-Silva, 2021, Th. 15) (for a more restricted family of classifiers). The proposed proof offers a more direct argument. Practical efficient algorithms for computing both AXp's and CXp's of DLs are described elsewhere (Ignatiev and Marques-Silva, 2021).

One final comment regarding DTs. There are polynomial time algorithms for computing CXp's (Huang et al., 2021b; Izza et al., 2022a), but also for enumerating all CXp's (Huang et al., 2021b; Izza et al., 2022a). Hence, for DTs, one can argue that there are efficient solutions to answering WHYNOT questions. However, the bookkeeping involved to prevent redundancy in reported CXp's (and so in answering WHYNOT questions) is arguably beyond the reach of the average human decision maker. The proposed algorithm (Huang et al., 2021b; Izza et al., 2022a) lists all possible ways to change a prediction (in polynomial time), and then removes the ways that exhibit redundancies (also in polynomial time). However, such algorithms would require a non-negligible amount of work if the solution were to be computed manually.

## 6. Experimental evidence

This section overviews the experimental results aiming to practically confirm the claims made earlier in the article. Namely, this section will assess the redundancy (as explained below) of the explanations offered “by default” by decision tree and decision list models trained with well-known and publicly available tools.

The experiments were built on the earlier results and data are published by Ignatiev and Marques-Silva (2021) and Izza et al. (2022a). The datasets and the induced DTs/DLs are adapted from these earlier works. In particular, for DTs, we use the SAT-based implementation of the explanation redundancy checker proposed in Izza et al. (2022a). For DLs, we use the SAT-based implementation of the formal explainer proposed in Ignatiev and Marques-Silva (2021) publicly available online^7^. The latter tool was augmented with the capability to measure redundancy of a given explanation as defined below.

The experiments were performed on a MacBook Pro laptop running macOS Ventura 13.0.1. Each individual process was run on a 6-Core Intel Core i7 2.60 GHz processor with 16 GB of memory. Despite the use of the 4 GB memory limit and 1800 s time limit, none of these limits has been reached for any of the problem instances used. In fact, the redundancy checkers were effective enough to stop and output a redundancy report *long before* the time limit.

As mentioned above, we considered the data from the earlier work. Therefore, all the datasets considered here are taken from the publicly available sources (Friedler et al., 2015; FairML, 2016; PennML, 2020; UCI, 2020) and are taken directly from Ignatiev and Marques-Silva (2021); Izza et al. (2022a). Following prior work (Izza et al., 2022a), we assessed the redundancy of explanations offered by decision trees trained by two prominent DT inference tools: ITI (Incremental Tree Induction) (Utgoff et al., 1997; ITI, 2020) and IAI (Interpretable AI) (Bertsimas and Dunn, 2017; IAI, 2020). When training IAI models, the tool was run being instructed to train high-accuracy DTs of depth either 6 and 8 (Deeper trees are harder to learn, and do not yield significant gains in accuracy). In the following, these two configurations of IAI are referred to as IAI-6 and IAI-8, respectively. As for decision list models, those were trained by the well-known CN2 algorithm (Clark and Niblett, 1989; Clark and Boswell, 1991).

### 6.1. Measuring explanation redundancy

Given a data instance, an explanation offered by a decision tree model “by default” is assumed to be a set of feature literals appearing in the path that fires the prediction for the instance. In the case of decision list models, a “default” explanation is constructed as the set of feature literals comprising the rule that fires the given prediction plus the first literal in each preceding rule that is determined to be inconsistent with the instance. Note that one does not have to always consider the first such literal. However, this strategy is simple enough be used by a human decision maker.

When a default explanation X is computed as detailed above, a redundancy checker is run to compute how many literals can be dropped from X resulting in an abductive explanation $X^{\prime }\subseteq X$, i.e., the AXp condition (4) holds for $X^{\prime }$. Afterwards, the redundancy of X is said to be equal to the portion of features in X that the redundancy check was able to remove, i.e., it is computed as the value of $100\%\cdot \frac{\left|X|-|X^{\prime }\right|}{\left|X\right|}$. Note that our experiment targets computing subset-minimal AXps $X^{\prime }$ rather than cardinality-minimal. In the latter case, the redundancy statistics of the default DT and DL explanations would clearly be even higher than the one reported below.

### 6.2. Redundancy of default explanations

**Figure 2:**
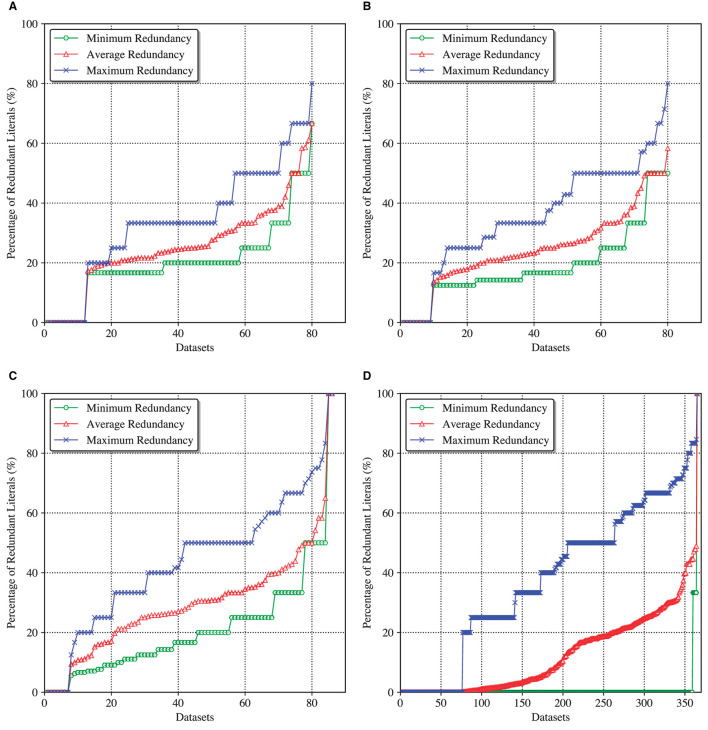
Minimum, average, and maximum redundancy of default DT and DL explanations. **(A)** Redundancy of DT/IAI6 explanations. **(B)** Redundancy of DT/IAI8 explanations. **(C)** Redundancy of DT/ITI explanations. **(D)** Redundancy of DL/CN2 explanations.

Figure 2 shows four cactus plots depicting the minimum, average, and maximum redundancy of default explanations computed for the considered DT and DL models. Here is how the plots should be interpreted. Given a dataset and the corresponding ML model, *each instance* of the dataset is provided with a default explanation by the model, as described above, which is followed by an explanation redundancy check. Given the redundancy information for all the instances of the dataset, the minimum, average, and maximum explanation redundancy for this dataset is calculated. As a result and considering all the datasets studied, the full minimum/average/maximum redundancy statistics is plotted as a line sorted in ascending order. This way, a point with coordinates (*X, Y*) signifies that there are *X* datasets/models whose default explanations have the (minimum, average, and maximum) literal redundancy upper-bounded by *Y%*.

As can be observed, with slight variation, all the models exhibit significant explanation redundancy. Although there are a large number of datasets whose instances are provided with irredundant default explanations (e.g., see zero minimum redundancy), some instances may receive explanations with 80%–100% redundant literals (see maximum redundancy) (Note that an explanation is 100% redundant if all the features can be removed from it; this occurs when the classifier computes a constant function). The most interesting information is associated with the average redundancy, calculated for each dataset across all of its instances. While the worst average redundancy is demonstrated by ITI's decision trees, it still reaches ≈ 60% for IAI's decision trees and ≈ 50% for CN2's decision lists. This means that for the corresponding datasets, on average 60% (50%, respectively) of literals can be dropped from a default explanation offered by an IAI decision tree (CN2 decision list, respectively).

These experimental results serve as evidence confirming the lack of *practical* interpretability of what is believed to be the most interpretable ML models. Consequently, they also demonstrate the need for computing irredundant and provably correct formal abductive (but also contrastive) explanations if interpretability and transparency of the decisions made by these models is of concern. It should also be noted that despite the use of the SAT technology dealing with NP-hard problems, the runtime of the redundancy checks applied in the experiment does not exceed a small fraction of a second per instance and can be neglected, which demonstrates that the proposed formal explainability approach is ready for widespread practical deployment.

### 6.3. Additional classifiers

Although the experiments reported in this article consider fairly shallow DTs (i.e., with depths not exceeding six or eight), which suffice in terms of target accuracy, the methods proposed in this article can be shown to apply for much larger (and deeper) DTs. For example, recent work (Ghiasi et al., 2020) proposes the use of DTs for diagnosis of coronary artery disease. For one of the DTs proposed in Ghiasi et al. (2020) (see Ghiasi et al., 2020, Figure 2), the longest paths have 19 non-terminal nodes. Among these, for the path with prediction cad, manual inspection^8^ reveals that at least 10 literals out of 19 (i.e., more than 50%) are redundant. Evidently, for a human decision maker, an explanation with nine literals (or less) is far easier to understand than an explanation with 19 literals.

## 7. Discussion

This article looks at so-called interpretable models from the perspective of explaining the predictions made. Explanations can serve to answer a WHY question, or alternatively a WHYNOT question. Recent work refers to the latter as abductive explanations and the latter as contrastive explanations (Ignatiev et al., 2019a, 2020a; Miller, 2019).

Because interpretable models are expected to serve themselves as the explanations (Rudin, 2019; Molnar, 2020), we focus on (manually) extracting answers to WHY questions from the models. In contrast, (manually) finding answers to WHYNOT questions is in general far less intuitive. In fact, Section 5.4 proves that it is computationally hard to answer WHYNOT questions for DLs. Clearly, all this but precludes human decision makers from attempting to answer WHYNOT questions for DLs.

Recent results (Izza et al., 2020, 2022a; Huang et al., 2021b) showed that, both in theory and in practice, decision trees exhibit explanation redundancy, i.e., if a path is used as the explanation for a WHY question, then that explanation exhibits redundancy when compared with a rigorous (logically-defined) explanation. More problematic, redundancy can grow arbitrarily large with path length.

The previous sections show that the same limitations occur with decision lists, and that decision sets exhibit other drawbacks that also serve to challenge its interpretability.

As shown by the experiments, the amount of redundancy in manually produced explanations, for DTs and DLs, can be significant. For many of the examples considered, the fraction of redundant literals exceeds 50%, i.e., more than one out of two literals in the explanation could be discarded, and that would not affect the correctness of the explanation.

Given the above, and as long as model comprehensibility is premised on succinctness, then neither decision trees, decision lists, or decision sets can be appropriate for (manually) answering WHY questions.

For WHYNOT questions, the situation is even more problematic. For DTs, CXp's (and so the answer to WHYNOT questions) can be computed in polynomial time, but such algorithms are beyond the reach of (the average) human decision makers. For DLs, it seems unrealistic to even ask a human decision maker to change a decision, since this problem is by itself computationally hard.

Given the results in this article, we conclude that answers to WHY and WHYNOT questions (or alternatively the computation of (rigorous) AXp's and CXp's) should be obtained with dedicated algorithms, as proposed in recent work (see Marques-Silva and Ignatiev, 2022 and references therein).

## 8. Conclusion and research directions

For high-risk application domains, there has been recent interest in so-called interpretable ML models (Lakkaraju et al., 2016; Rudin, 2019, 2022; Molnar, 2020). This article proposes model comprehensibility as a measure of the understanding of ML model predictions by human decision makers. Model comprehensibility aims at finding explanations, i.e., answers to WHY and WHYNOT questions, which are both correct and irreducible. The motivation is that, for interpretable models, one would expect predictions to be comprehensible by human decision makers. As argued in this article, it is hardly the case that existing interpretable models can be deemed to enable model comprehensibility. Hence, even though there are a number of valid reasons to deploy interpretable models in high-risk domains, the ability to find correct and irreducible explanations, by manual inspection, is not among them. Despite the fact that one can identify general rules that allow for a human decision maker to find correct explanations by inspection, it is also the case that such explanations can in general be arbitrarily redundant on the number of features. The solution for this limitation of interpretable models is, as it is also the case with non-interpretable models, to compute rigorous explanations. Moreover, it is the case that rigorous explanations can be (very) efficiently computed for both decision trees and decision lists.

Furthermore, and although decision sets can also be easily explained in practice (Ignatiev and Marques-Silva, 2021), it is also the case that most publicly available solutions for the creation of decision sets exhibit a number of crucial drawbacks. One example is prediction overlap; another is the need to use a default rule for when no other rule fires. Both drawbacks represent critical limitations to model comprehensibility.

## Data availability statement

Publicly available datasets were analyzed in this study. This data can be found at: https://blog.fastforwardlabs.com/2017/03/09/fairml-auditing-black-box-predictive-models.html; https://github.com/EpistasisLab/pmlb; https://archive.ics.uci.edu/ml.

## Author contributions

All authors listed have made a substantial, direct, and intellectual contribution to the work and approved it for publication.

## Footnotes

^1^Extraction of rules from decision trees has been studied before (Quinlan, 1987), but not for addressing explaination redundancy.

^2^PI explanations were first proposed in the context of boolean classifiers based on restricted bayesian networks (Shih et al., 2018). Independent work (Ignatiev et al., 2019a) studied PI explanations in the case of for more general classification functions, i.e., not necessarily boolean, and related instead explanations with abduction. This article follows the formalizations used in more recent work (Marques-Silva et al., 2020, 2021; Cooper and Marques-Silva, 2021; Huang et al., 2021b, 2022; Ignatiev and Marques-Silva, 2021; Izza and Marques-Silva, 2021; Ignatiev et al., 2022; Marques-Silva and Ignatiev, 2022).

^3^Each predicate associated with a given concept will be noted in sans-serif letterform. When referring to the same concept in the text, the same acronym will be used, but in standard letterform. For example, the predicate name AXp will be used in logic statements, and the acronym AXp will be used throughout the text.

^4^In this article, contrastive explanations mimic counterfactual explanations. However, in more complex explanation scenarios, they may differ (Liu and Lorini, 2022b).

^5^Following notation that is standard in theoretical computer science, computational problems that are NP-hard are deemed theoretically intractable; however, there might exist practical algorithms that are efficient in practice, albeit not in the worst case. In contrast, polynomial-time algorithms are always assumed to be efficient in practice.

^6^As clear from the outset, we are interested in rigorous explanations to predictions, i.e., explanations that are both correct, given some agreed definition of correct, and irreducible.

^7^https://github.com/alexeyignatiev/xdl-tool.

^8^Unfortunately, we have been unable to obtain from the authors this concrete DT in a format suitable for automated analysis.
